# A T/C polymorphism in the *GPX4* 3′UTR affects the selenoprotein expression pattern and cell viability in transfected Caco-2 cells

**DOI:** 10.1016/j.bbagen.2011.03.016

**Published:** 2011-06

**Authors:** Hannah Gautrey, Fergus Nicol, Alan A. Sneddon, Judith Hall, John Hesketh

**Affiliations:** a Institute for Cell and Molecular Biosciences, Newcastle University, Framlington Place, Newcastle upon Tyne, NE2 4HH, UK; b Human Nutrition Research Centre, The Medical School, Newcastle University, Framlington Place, Newcastle upon Tyne, NE2 4HH, UK; c Rowett Institute of Nutrition and Health, University of Aberdeen, Greenburn Rd, Bucksburn, Aberdeen, AB21 9SB, UK

**Keywords:** Selenium, Glutathione peroxidase 4, Oxidative stress, 3′untranslated region, Selenoprotein hierarchy, Transfection

## Abstract

**Background:**

Synthesis of selenoproteins such as glutathione peroxidases (GPx) requires a specific tRNA and a stem-loop structure in the 3′untranslated region (3′UTR) of the mRNA. A common single nucleotide polymorphism occurs in the *GPX4* gene in a region corresponding to the 3′UTR.

**Methods:**

The two variant 3′UTR sequences were linked to sequences from a selenoprotein reporter gene (iodothyronine deiodinase) and expressed in Caco-2 cells. Clones expressing comparable levels of deiodinase (assessed by real-time PCR) were selected and their response to tert-butyl hydroperoxide assessed by cell viability and measurement of reactive oxygen species. Selenoprotein expression was assessed by real-time PCR, enzyme activity and immunoassay.

**Results:**

When selenium supply was low, cells overexpressing the C variant 3′UTR showed lower viability after oxidative challenge, increased levels of reactive oxygen species and lower GPx activity and SelH mRNA expression compared to cells overexpressing the T variant. After selenium supplementation, cell viability and GPx4 expression were higher in the cells overexpressing the C variant. Expression of transgenes incorporating the T/C variant *GPX4* (rs713041) sequences in Caco-2 cells leads to alterations in both cell viability after an oxidative challenge and selenoprotein expression. This suggests that the two variants compete differently in the selenoprotein hierarchy.

**General Significance:**

The data provide evidence that the T/C variant *GPX4* (rs713041) alters the pattern of selenoprotein synthesis if selenium intake is low. Further work is required to assess the impact on disease susceptibility.

## Research highlights

► Clones over-expressed a GPX4 3′UTR transgene with either T or C variant of rs713041. ► T/C polymorphism modulated effect of over-expression on selenoprotein hierarchy. ► T/C polymorphism modulated effect of transgene on sensitivity to oxidative challenge.

## 1 Introduction

There is increasing evidence that genetic variants such as single nucleotide polymorphisms (SNPs) can alter nutrient metabolism and responses to changes in nutrient supply [1,2]. The interaction between such functional genetic variants and diet is likely to underlie many multifactorial diseases [e.g. 3]. Sub-optimal intake of the micronutrient selenium (Se) has been associated with increased risk of cardiovascular disease and prostate and colorectal cancers [4,5]. The metabolic functions of Se are thought to be due to its presence as selenocysteine in ~ 25 selenoproteins in mammals. Many of these proteins, including the glutathione peroxidases (GPx) and thioredoxin reductases (TR), are thought to have antioxidant and redox functions and protect cells from oxidative stress and cell damaging agents [6–8]. As a result, SNPs in selenoprotein genes may be expected to affect antioxidant functions.

Se is incorporated into selenoproteins during protein synthesis using UGA as the codon for selenocysteinyl-tRNA; this requires a selenocysteine insertion sequence (SECIS) in the 3′untranslated region (3′UTR) of the mRNAs and specific proteins that bind to these RNA structures [6]. When Se supply is limiting there is a tissue-specific prioritisation of selenoprotein synthesis so that synthesis of certain proteins is maintained at the expense of synthesis of others [6,9] and this leads to differential effects of Se supply on the levels of both the selenoproteins and their corresponding mRNAs. This prioritisation, often referred to as the selenoprotein hierarchy, is partly dependent on differences in the ability of the different 3′UTR sequences to interact with the selenocysteine incorporation machinery [6,9–11]. As a result, SNPs in the selenoprotein gene regions corresponding to the 3′UTR could have functional effects, potentially affecting not only the synthesis of the selenoprotein coded by the gene containing the SNP but also the synthesis of other selenoproteins.

A T/C variation (rs713041) in the region of the *GPX4* gene that corresponds to the 3′untranslated region (3′UTR) of the mRNA has been found in Caucasian and Asian populations [12,13]. Using a transfected cell model expressing a selenoprotein iodothyronine deiodinase (IDI) reporter gene the variants have been shown to drive selenoprotein synthesis to different extents [14]. Furthermore, results from a human supplementation trial suggest that this SNP affects expression of lymphocyte GPx1 and GPx4, and *in vitro* assays with Caco-2 cell extracts indicate that the T and C variants of the 3′UTR show different protein binding characteristics [15], suggesting that the variants differ in their ability to interact with the Se incorporation machinery. However, it is not known if the SNP affects either the cells' ability to respond to an oxidative challenge or the hierarchy of selenoprotein synthesis.

The aim of the present work was to investigate whether the T and C variants of this SNP differ in their ability to affect these parameters. To do this we produced stable clones of transfected Caco-2 cells over-expressing comparable amounts of transcripts encoding the selenoprotein iodothyronine deiodinase linked to the *GPX4* 3′UTR containing either T or C variant. These transfected cells were used to assess the impact of the presence of T and C variant transcripts on selenoprotein expression and response to a *tert*-butylhydroperoxide challenge. The results are consistent with over-expression of transcripts containing the C variant of rs713041 having a greater effect on the selenoprotein hierarchy than the T variant.

## 2 Methods

### 2.1 Cell culture

Caco-2 cells were maintained in Dulbecco's minimal Eagle's medium (DMEM) containing 10% foetal calf serum and penicillin and streptomycin. Cells were incubated at 37 °C with 5% CO_2._ For growth of cells in a specific concentration of selenium, cells were grown in serum free DMEM with added insulin (5 μg/ml) and transferrin (5 μg/ml) and varying concentrations of sodium selenite, as described previously [16]. Caco-2 cells are heterozygous C/T for rs713041.

### 2.2 DNA transfection

The construction of the pCDNA3.1/Zeo (+) plasmids containing the coding region of rat type 1 iodothyronine deiodinase (IDI) with the 3′UTR of *GPX4* containing either a T (IDI-GPX4(T)) or a C (IDI-GPX4(C)) at position 718 was described previously [14]. Caco-2 cells were transfected at 90–95% confluency with endotoxin-free IDI-GPX4(T) or IDI-GPX4(C) plasmids (μg) using Lipofectamine 2000 reagent (μl) (Invitrogen) in a 1:3.5 ratio according to the manufacturer's instructions. After 24 hours, the cells were split (1:5) and grown for an additional 24 hours in normal media. Cells were then grown in a selective media containing 200 μg/ml of the antibiotic zeocin and stably transfected colonies isolated for both IDI-GPX4(T) or IDI-GPX4(C) transfected cells. Two IDI-GPX4(T) and two IDI-GPX4(C) clones were selected for use in further experiments based on their IDI expression levels.

### 2.3 Cell viability assays

Ninety-six well plates were seeded with 6 × 10^4^ cells/well, and after 24 hours half the cells were treated with varying concentrations of *tert*-butylhydroperoxide diluted in media. After 4 hours the medium containing *tert*-butylhydroperoxide was removed and fresh medium added to the cells. Cell viability was assessed by the addition of 20 μl of CellTiter-Blue^®^ reagent (Promega) and after 4 hours absorbance was measured at 560 nm.

### 2.4 ROS measurement

Cells were seeded at a density of 3 × 10^4^ cells/well in 96 well plates and grown for 2 days. To induce ROS production, cells were incubated for 4 hours with 100 μM *tert*-butylhydroperoxide, following instructions from the manufacturers of the detection kit. Cells were washed with Hanks' balanced salt solution without phenol red (HBSS; from Invitrogen) before incubation with 25 μM 5-(and-6)-carboxy-2′,7′-dichlorodihydrofluorescein diacetate (carboxy-H_2_DCFDA (Invitrogen)) and 0.5 μM Hoechst 33342 dye diluted in HBSS . After 15 minutes incubation the cells were then washed twice with HBSS after which 50 μl of HBSS was added to each well. Florescence of the two dyes was measured by excitation at 485 nm and detection of emitted fluorescence at 520 nm for carboxy-H_2_DCFDA, and by excitation at 355 nm and detection of emitted fluorescence at 460 nm for Hoechst 33342.

### 2.5 RT-PCR

Cells were washed with phosphate buffered saline before total RNA was extracted with Trizol (Invitrogen) and purified using PureLink™ columns (Invitrogen). cDNA was synthesised at 55 °C for 30 minutes from 1 μg of total RNA using Oligo-dT primers and transcriptor reverse transcriptase (Roche). Endogenous mRNA levels were measured on equivalent amounts of total RNA by real-time using a Roche LightCycler 480 and SYBR Green PCR master mix (Roche). Primers used for the real time PCR were as follows:

**Table:** 

hGPX1	Forward 5′-GAGAATGTGGCGTCCCTCT-3′
	Reverse 5′-CTCTTCGTTCTTGGCGTTCT-3′
hGPX4	Forward 5′-AGACCGAAGTAAACTACACTCAGC-3′
	Reverse 5′-CGGCGAACTCTTTGATCTCT-3′
hTxnrd1	Forward 5′-TGGAACTAGATGGGGTCTCG-3′
	Reverse 5′-CTTAACTGTCTCCTCGACTTTCCAT-3′
hSelH	Forward 5′-CTTCGAGGTGACGCTGCT-3′
	Reverse 5′-CTTGAGGCTCAGGGAATTTG-3′
rIDI	Forward 5′-GGTGGCTACGGGCAAGGTGC-3′
	Reverse 5′-GGACCCAGTTGTCAGGGGCG-3′
GAPDH	Forward 5′-TGAAGGTCGGAGTCAACGGATTTG-3′
	Reverse 5′-CATGTAAACCATGTAGTTGAGGTC-3′

The thermal profile for PCR consisted of 94 °C for 2 minutes, 35 cycles of 94 °C for 30 seconds, 59 °C for 45 seconds, 72 °C for 1 minute and finally 2 minutes at 72 °C. Samples and negative controls were analysed in triplicate and the amount of mRNA normalised to GAPDH mRNA. Relative quantification was carried out from a standard curve using dilutions of purified PCR products obtained with appropriate primer sets.

### 2.6 Selenoprotein assays

Caco-2 cell extracts were prepared by resuspending cell pellets into 0.1% Triton (peroxide-free) in PBS, sonication for 10 seconds and centrifugation at 3500*g* for 10 minutes. GPx activity in the supernatant fluid was determined by the method of Paglia and Valentine as modified by Brown et al. using hydrogen peroxide as a substrate [17]. One unit of GPx activity is defined as that which oxidises 1 μmol of NADPH/min. GPx4 protein levels were measured by a competitive ELISA [18] using a rabbit polyclonal antibody (raised against the whole human recombinant GPx4 protein) and human recombinant GPx4 protein (both LabFrontier (Seoul, Korea)). The ELISA was performed in 96-well plates that were coated with the polyclonal anti-human GPX4 antibody at 1:10,000 dilution. The ELISA used the principle of competitive binding whereby calibrator/sample and biotinylated human GPX4 competed for binding to the GPX4 antibody-coated wells. For the signal reagent, NeutrAvidin (Perbio, Cramlington, UK) horseradish peroxidase diluted 1:10,000 in 0.5% casein was added to each well and incubated at 37 °C for 1 hour. The plates were then washed three times with PBS/Tween. Tetramethylbenzidine was added to each well and incubated at room temperature for up to 15 minutes. The reaction was stopped with 0.18 M of H_2_SO_4_ and the absorbance read at 450 nM. TR1 protein levels were also measured by competitive ELISA [18] using rabbit anti-TR1 antiserum (a gift from Dr Forbes Howie, University of Edinburgh) at a final dilution of 1:30,000 and recombinant TR1 protein obtained from Labfrontier (Seoul, Korea). TR1 protein was biotinylated using a commercial kit (Sigma, UK) and a calibration curve prepared over a range of concentrations (0.3–40 ng/ml).

## 3 Results

Caco-2 cells do not express deiodinase at a level detectable by enzyme assay [14]. Following transfection of Caco-2 cells with rat IDI coding sequences linked to the *GPX4* 3′UTR with either the T (IDI-T) or C (IDI-C) variant corresponding to rs713041, IDI transcripts were detected by semi-quantitative RT-PCR and real-time PCR using primers specific to rat IDI that did not detect human IDI sequences in untransfected Caco-2 cells (results not shown). For both variants there was an up to 10-fold range of expression of the transgene between the various clones. Since the two variants differ only in the 3′UTR, assessment of their relative effects was carried out in clones selected on the basis that they expressed IDI-T and IDI-C transcripts at comparable levels. In the first series of experiments two clones expressing IDI-T and two expressing IDI-C transcripts at similar levels were selected and the mean IDI transcript levels (as arbitrary units relative to GAPDH) in the clones studied were 0.51 ± 0.1 and 0.30 ± 0.1 for the IDI-T and IDI-C cells respectively.

**Fig. 1:**
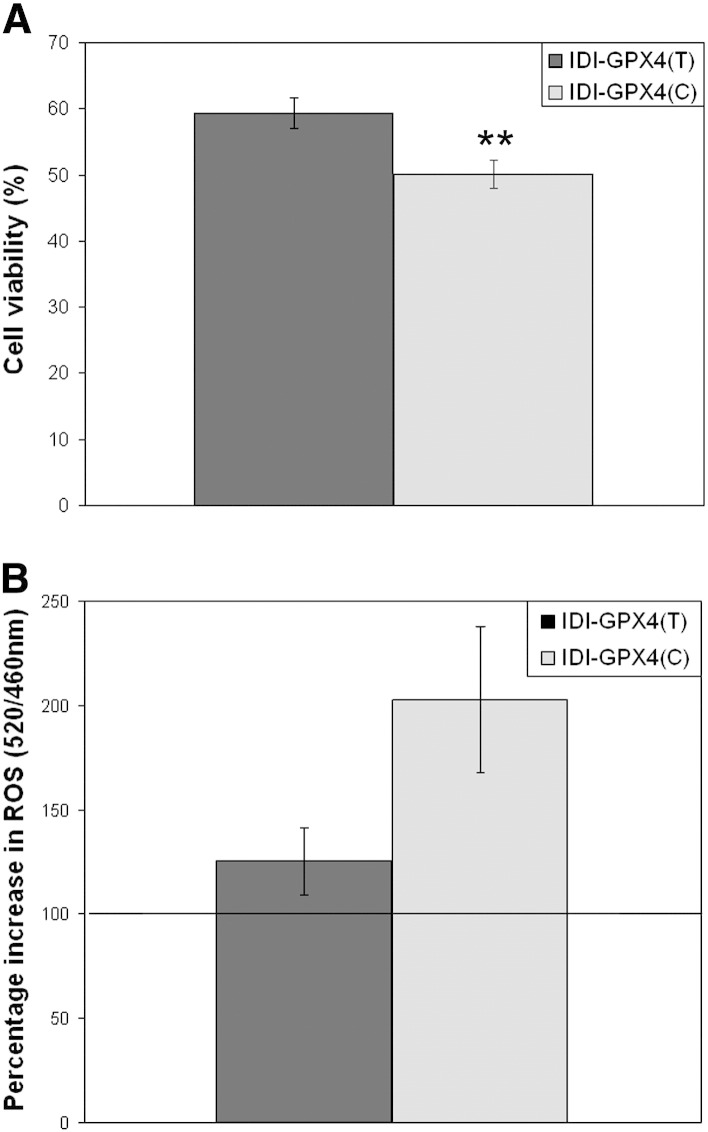
Cell viability and ROS levels in response to the oxidant *tert*-butylhydroperoxide. (A) Percentage viability of the IDI-T and IDI-C clones after 4 hours of treatment with 3 mM *tert*-butylhydroperoxide compared to untreated IDI-T and IDI-C clones. (B) Percentage increase in ROS after 4 hours of treatment with 100 μM *tert*-butylhydroperoxide compared to untreated cells. All cells were grown in normal serum containing media. Values shown are means ± s.e.m.

Overall cell viability of the cells was assessed after an oxidative challenge without distinguishing between necrotic and apoptotic cell death. In order to allow us to carry out such experiments without lengthening periods of altered Se supply, viability was measured within hours of the challenge. When grown in normal medium untransfected Caco-2 cells showed considerable resistance to *tert*-butylhydroperoxide and a concentration of 3 mM was chosen as the challenge since it led to approximately 50% cell viability under these conditions. In Se-depleted medium a lower concentration of *tert*-butylhydroperoxide was used as the challenge because of the additional sensitivity of the cells. Viability was measured in cells grown in normal culture medium 4 hours after challenge with *tert*-butylhydroperoxide. In unchallenged conditions, cells over-expressing either IDI-T or IDI-C transcripts showed lower cell viability compared with untransfected cells (data not shown). The transfected cells showed lower viability over a range of *tert*-butylhydroperoxide concentrations with 40–60% viability when challenged with 1–5 mM *tert*-butylhydroperoxide. The fall in cell viability after a challenge with 3 mM *tert*-butylhydroperoxide was significantly greater in cells expressing IDI-C compared with those expressing the IDI-T (*p* = 0.006, Fig. 1A). One explanation of this effect is that the T and C variant 3′UTRs compete for available Se and selenocysteine incorporation machinery to different extents and so divert Se from synthesis of antioxidant selenoproteins to synthesis of the IDI transgene (which has no antioxidant function) differentially. To test this, measurements of ROS and selenoprotein levels were carried out.

**Fig. 2:**
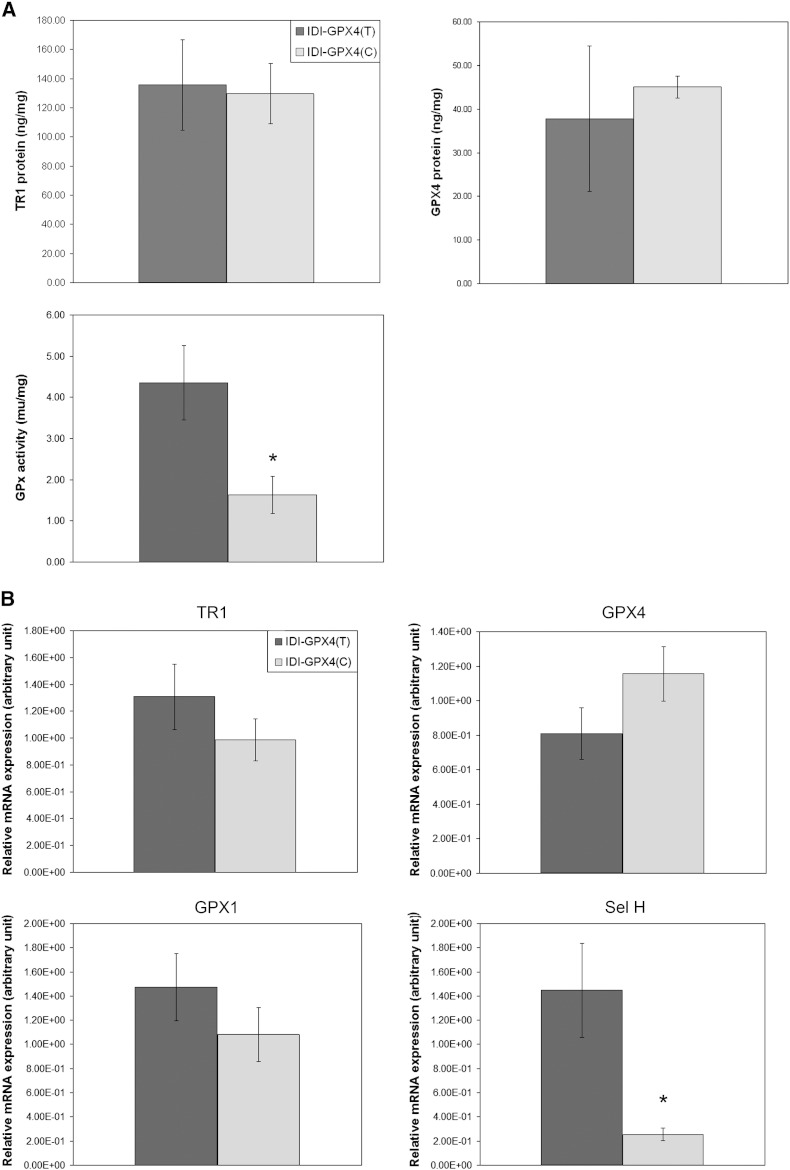
Selenoprotein expression in clones expressing IDI-C and IDI-T. (A) Levels and activity of selenoproteins in the IDI-T and IDI-C clones grown in normal serum containing medium. TR1 and GPx4 protein levels were measured by ELISA and normalised by total protein. GPx activity was measured and normalised by total protein content. (B) Real time PCR analysis of the IDI-T and IDI-C clones grown in normal serum containing media. Gene expression was measured for the selenoproteins TR1, GPx4, GPx1 and SelH and normalised by GAPDH (ratio). Values shown are means ± s.e.m.

In order to measure ROS levels following *tert*-butylhydroperoxide treatment it was necessary to use a lower concentration of the oxidant. Challenge of the cells with 100 μM *tert*-butylhydroperoxide led to measurable increases in total reactive oxygen species (ROS) after 4 hours but retained cell viability. When IDI-C and IDI-T cells were grown in normal culture medium and challenged with *tert*-butylhydroperoxide there was a greater increase in levels of ROS in IDI-C cells compared with IDI-T cells (*p* = 0.053; Fig. 1B) and this difference suggested that the IDI-C cells were under greater oxidative stress. When grown in normal medium the IDI-C cells showed a significantly lower GPx activity compared with the IDI-T cells but levels of TR1 and GPx4 were similar (Fig. 2A). Real-time PCR showed mean levels of TR1, GPx1 and GPx4 mRNAs to be similar in the IDI-T and IDI-C cells but levels of selenoprotein H mRNA were significantly lower in IDI-C cells compared with IDI-T (Fig. 2B).

**Fig. 3:**
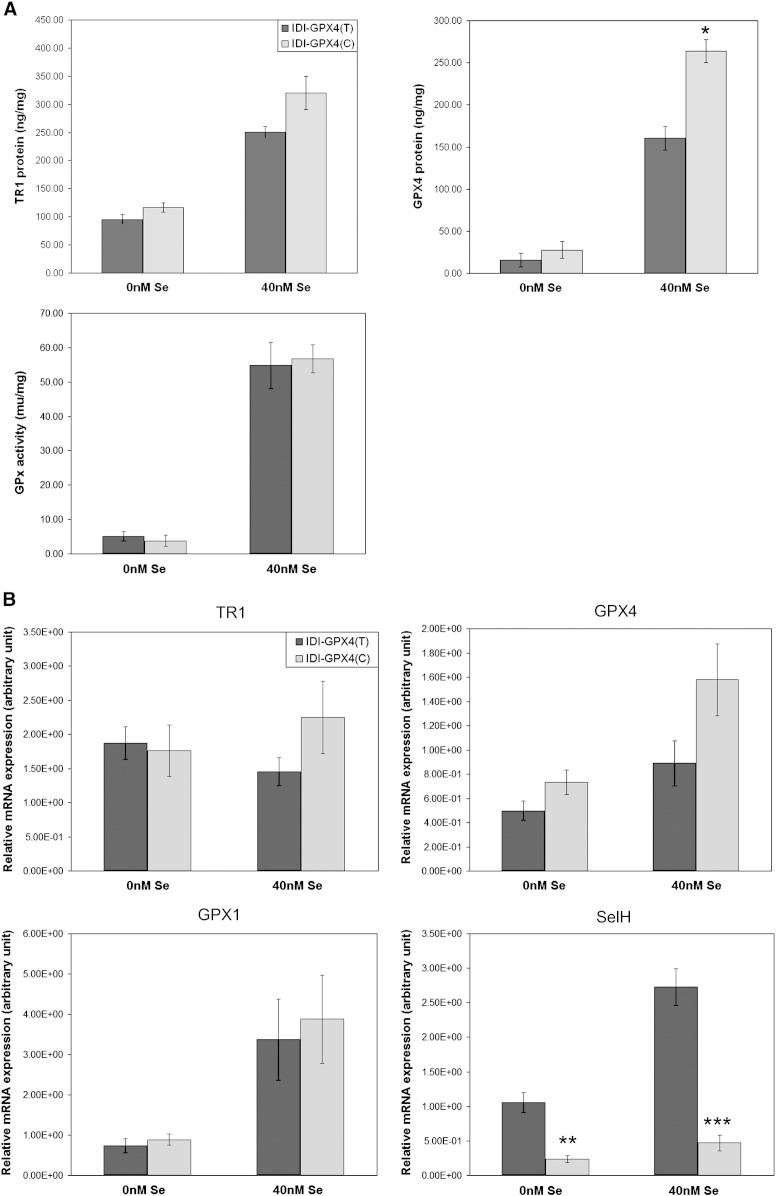
Selenoproteins in clones expressing IDI-C and IDI-T grown in selenium deficient and sufficient media. Cells were grown in selenium deficient (0 nM Se) or selenium sufficient (40 nM Se) media for 3 days prior to collection of protein and total RNA. (A) Protein levels of TR1 and GPx4 measured by ELISA and GPx activity, all normalised by total protein. (B) Level of selenoprotein mRNA measured by real-time PCR and normalised by GAPDH (ratio). Values shown are means ± s.e.m.

To assess the impact of the T/C variants on the selenoprotein hierarchy under conditions of different Se supply, measurements of selenoprotein expression were made in clones grown in Se-depleted and Se-supplemented media. As shown in Fig. 3, in both sets of transfected cells GPx activity, TR1 protein and GPx4 protein levels were 80–90% lower in Se-depleted compared with Se-supplemented cells. Notably the levels, particularly for GPx activity, were similar in cells grown in Se-depleted and cells grown in normal media (DMEM with 10% serum), and we speculate that since GPx1 activity is a sensitive marker of Se status [10,20] the normal medium used in these experiments was low in Se [21]. In Se-depleted conditions there was no significant difference in GPx activity, TR1 protein or GPx4 protein levels between ID1-C and IDI-T cells. When the cells were grown in Se-supplemented medium the IDI-C cells showed a higher content of GPx4 protein compared with the IDI-T cells (Fig. 3). This suggests that when sufficient Se is present there is a greater increase in GPx4 in IDI-C cells*.* Real-time PCR showed mean levels of TR1, GPx1 and GPx4 mRNAs to be similar in the IDI-T and IDI-C cells grown in either Se-depleted or supplemented medium but levels of selenoprotein H mRNA were significantly lower in IDI-C cells compared with IDI-T whether the cells were grown in either Se-depleted or supplemented medium (Fig. 3).

**Fig. 4:**
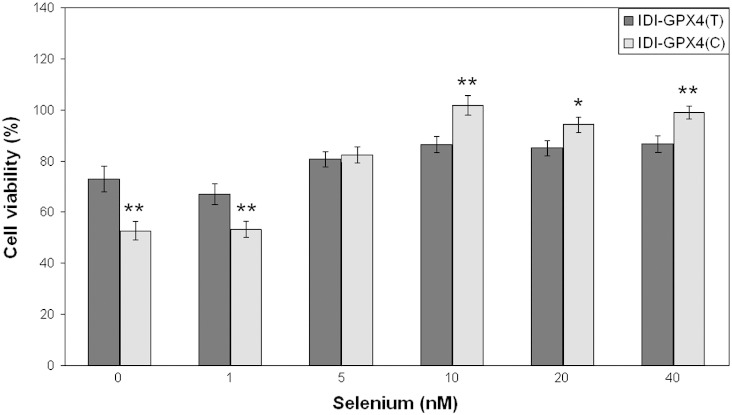
The effect of selenium on cell viability. IDI-T and IDI-C clones were grown for 3 days in serum free media containing varying concentrations of selenium (0, 1, 5, 10, 20 and 40 nM). Cell viability was measured after 4 hours treatment with 0.5 mM *tert*-butylhydroperoxide and is shown as a percentage of untreated IDI-T and IDI-C clones grown in the same concentration of selenium. Values shown are means ± s.e.m.

Since selenoprotein expression appeared different in IDI-C and IDI-T cells depending on the Se supply, experiments were carried out to assess how the Se content of the medium affected cell viability following challenge with *tert*-butylhydroperoxide. In these experiments, where cells were grown under serum-free conditions to deplete Se, cells were more sensitive to the oxidant and so 0.5 mM *tert*-butylhydroperoxide was used as the challenge. As shown in Fig. 4 in Se-depleted medium IDI-C cells were less viable than the IDI-T cells and this was also the case when a very low concentration of Se (1 nM) was added to the medium. As the Se concentration was increased further there was no difference in viability at 5 nM Se and then at higher concentrations of Se (10–40 nM) the difference in viability was reversed and IDI-C cells showed greater viability than IDI-T after the *tert*-butylhydroperoxide challenge. These data suggest that when Se supply is low IDI-C cells are under greater oxidative stress than IDI-T cells after a challenge with *tert*-butylhydroperoxide but that this is not the case when cells are grown in Se supplemented medium when IDI-C cells show greater viability.

**Fig. 5:**
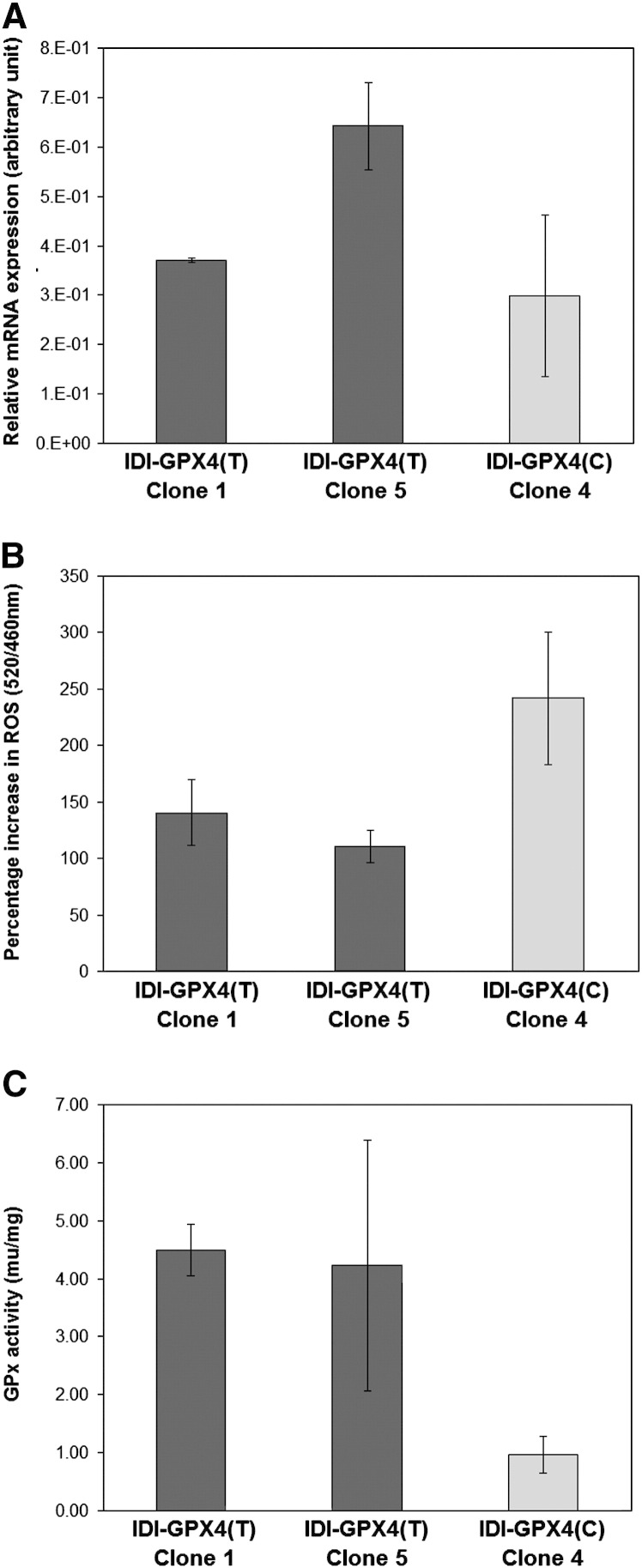
ROS levels and GPx activity in selected IDI-T and IDI-C clones. Individual clones were selected on the basis of IDI mRNA expression (A); two IDI-T clones were selected that expressed comparable or increased levels of the IDI transgene compared with an IDI-C clone. (B) ROS levels after challenge with 100 μM *tert*-butylhydroperoxide were not statistically different in the two T clones but were significantly greater in the IDI-C clone than in the T5 clone. (C) GPx activity was not statistically different in the two T clones but was significantly lower in the IDI-C clone compared with the T1 clone. Values shown are means ± s.e.m.

Finally, to exclude the possibility that the observed differences in ROS and selenoprotein expression could have been due to differences in levels of expression of the transgene, effects on single clones were compared. First, ROS levels were measured in IDI-T and IDI-C clones expressing comparable mean IDI transcript levels (0.37 and 0.30, as arbitrary units relative to GAPDH) and exposed to 100 μM tert-butylhydroperoxide. As shown in Fig. 5, mean ROS levels showed a greater increase in the IDI-C clone (2.4-fold) than in IDI-T clones (1.4 fold) and the difference between clone T5 and C4 was statistically significant (*p* < 0.05). In contrast two IDI-T clones showing a 2-fold difference in IDI expression (0.37 and 0.64, as arbitrary units relative to GAPDH) showed no statistically significant difference in response to tert-butylhydroperoxide. Second, the two IDI-T clones showing differences in IDI expression showed no significant difference in GPx activity whereas the IDI-T and IDI-C clones expressing similar levels of the transgene showed marked difference in mean GPx activity, with the difference between clone C4 and T1 being statistically significant (*p* = 0.023). These data support those in Figs. 1–3 in indicating that expression of IDI-C has a greater impact on endogenous GPx activity and ROS levels in response to an oxidative challenge.

## 4 Discussion

Previous work has suggested that a T/C variation (rs713041) in the region of the *GPX4* gene that corresponds to the 3′untranslated region (3′UTR) of the mRNA has functional consequences [12–15]. The variants have been shown to lead to transcripts with different protein binding properties, to drive selenoprotein synthesis to different extents and to alter lymphocyte GPx1 and GPx4 expression *in vivo*. The data from the present work extend these observations suggesting that this SNP is functionally significant by providing evidence that over-expression of transcripts containing the *GPX4* 3′UTR with either the T (IDI-T) or C (IDI-C) variant leads to altered selenoprotein expression and sensitivity of Caco-2 cells to oxidative challenge and Se-depletion.

Two observations suggest that the IDI-C cells were more sensitive to an oxidative challenge. First, measurement of ROS levels after challenge with *tert*-butylhydroperoxide showed a greater increase in IDI-C cells compared with IDI-T cells (Figs. 1B and 5), indicating that the IDI-C cells are under greater oxidative stress. Second, cells expressing the IDI-C variant showed a greater fall in cell viability after a challenge with *tert*-butylhydroperoxide compared with those expressing the IDI-T. Together these observations indicate that not only are the cells expressing the IDI-C transgene under greater oxidative stress but that the T and C variants at rs713041 in the *GPX4* 3′UTR have a differential impact on cell function. Furthermore, measurements of viability in cells grown in different Se concentrations showed that the effect of the IDI transgene on cell viability was modulated by Se supply, with IDI-C cells being more susceptible than IDI-T cells to oxidative challenge when Se-depleted but less susceptible when the Se supply was adequate. These data suggest that when Se supply is low IDI-C cells are under greater oxidative stress than IDI-T cells after a challenge with *tert*-butylhydroperoxide but that this is not the case when cells are grown in Se supplemented medium when IDI-C cells show greater viability. One explanation of these effects is that the T and C variant 3′UTRs compete for available Se and selenocysteine incorporation machinery to different extents and so divert Se for synthesis of the IDI transgene (which has no antioxidant function) differentially. Our hypothesis is that this then alters the balance of expression of endogenous selenoproteins that have redox and antioxidant functions.

Selenoprotein levels and expression of selenoprotein mRNAs have both been found to respond to Se supply and the effects differ between selenoproteins [6,9], leading to the concept of the selenoprotein hierarchy in which the level of selenoproteins and their mRNAs, and mRNA stability, are differentially affected by Se supply. GPx1 synthesis is known to be very sensitive to Se supply and GPx1 is low in the selenoprotein hierarchy in many cell types [6,9,20] including Caco-2 cells [16]. In comparison, GPx4 and TR1 are high in the hierarchy and less affected by Se depletion. GPx1, TR1 and GPx4 are key selenoproteins that react with ROS and regulate redox state [6]. SelH mRNA levels are highly sensitive to Se intake in the mouse colon [19] suggesting it is also low in the hierarchy. In the present experiments measurement of GPx1, GPx4, TR1 protein and mRNA levels, as well as SelH mRNA expression, indicated that IDI-C cells have lower expression of two selenoproteins (GPx1 and SelH) that are low in the hierarchy and sensitive to Se supply. This suggests that the T and C variant of the *GPX4* 3′UTR compete differently in the selenoprotein hierarchy with the C variant competing more strongly.

The impact of the transgene on selenoprotein expression was affected by Se supply, with GPx activity being lower in IDI-C cells than IDI-T cells when cells were grown in normal medium but this difference was not observed when cells were grown in Se supplemented medium. However, GPx activity was similar in Se-depleted IDI-C and IDI-T cells. The explanation for this is not clear but may reflect an effect of the insulin or transferrin present in the low Se medium compared with that of foetal calf serum in normal medium.

In addition, IDI-C and IDI-T cells showed similar levels of GPx4 protein when grown in normal or Se-depleted medium but the IDI-C cells had a higher GPx4 level when medium was supplemented with Se. When grown in medium supplemented with Se, IDI-C cells also showed greater cell viability after oxidative challenge and this may be due to the increased GPx4 protein levels in the IDI-C cells. We hypothesise that when the transfected cells are grown in an adequate Se supply, synthesis of selenoproteins high in the hierarchy (e.g. GPx4) is able to compensate for the reduced synthesis of those lower in the hierarchy, such as SelH and GPx1.

In summary, the present results indicate that expression of transgenes incorporating the T/C variant *GPX4* (rs713041) sequences in Caco-2 cells leads to alterations in both cell viability and ROS levels after an oxidative challenge and to changes in selenoprotein expression. Previous work has shown that this T/C variation in the 3′UTR of *GPX4* has altered RNA-protein binding properties and an altered ability to promote selenoprotein synthesis [14,15]. The present findings complement this earlier work by providing further evidence that rs713041 is an SNP with functional consequences and by indicating that its effects include alterations in the pattern of selenoprotein expression. Binding of the different selenoprotein 3′UTRs to proteins involved in selenoprotein synthesis is thought to be a major factor in determining the pattern of selenoprotein synthesis and how this changes with Se supply, so leading to the selenoprotein hierarchy [6]. Further experiments are needed to define how the SNP affects this selenoprotein hierarchy and overall selenoprotein function in the colonic epithelial cell and to define its impact on disease susceptibility.
